# How to Conquer Pain

**Published:** 1898-11

**Authors:** 


### How to Conquer Pain.

THE IMAGINATION OFTEN CONTROLS EVEN THE SUFFERINGS OF A BABY.

As every physician will testify, pain is greatly intensified by the imagination, and half of our suffering would be obviated if we had no previous knowledge of the curious sensation which we have called by that name.

"I am teaching my baby not to know that there is such a thing as pain," said a young mother, brightly and confidently.

"If he hurts himself and puckers up his face to cry we laugh at him, and then he will always laugh, too, even though I know the place must be smarting." Of course, such education has its obvious limitations, but there is a great deal in the theory, nevertheless. If baby were commisserated with instead of laughed at he would undoubtedly cry lustily. A fine, healthy setter dog who would come back torn and bleeding, but uproariously happy, after a successful canine fight, could be made to howl with anguish by its owner when he had no physical ill whatever, simply by the latter adopting a commisserating tone and pitying him for imaginary woes.

"Oh, the poor foot," his mistress would say. "How did Jowler get it hurt?" And the dog would lift up his paw, look at it mournfully, and, if the sympathy were continued, would commence to lick it and moan and whine as if he were in the greatest suffering, believing evidently that he felt all the pain that he imagined he was feeling.

The suffering that we dread at a dentist's would be a mere nothing if it came accidentally and without forethought, and with a severe headache sleep causes oblivion. Why should we not, therefore, merely consider pain as simply a peculiar sensation, and, unless particularly severe, as not involving suffering?

